# Exogenous d‐β‐hydroxybutyrate lowers blood glucose in part by decreasing the availability of L‐alanine for gluconeogenesis

**DOI:** 10.1002/edm2.300

**Published:** 2021-11-16

**Authors:** Adrian Soto‐Mota, Nicholas G. Norwitz, Rhys D. Evans, Kieran Clarke

**Affiliations:** ^1^ Department of Physiology, Anatomy and Genetics University of Oxford Oxford UK

**Keywords:** alanine, diabetes, d‐β‐hydroxybutyrate, exogenous ketosis, gluconeogenesis, hyperglycaemia, hypoglycaemia, ketone bodies

## Abstract

**Background:**

Interventions that induce ketosis simultaneously lower blood glucose and the explanation for this phenomenon is unknown. Additionally, the glucose‐lowering effect of acute ketosis is greater in people with type 2 diabetes (T2D). On the contrary, L‐alanine is a gluconeogenic substrate secreted by skeletal muscle at higher levels in people with T2D and infusing of ketones lower circulating L‐alanine blood levels. In this study, we sought to determine whether supplementation with L‐alanine would attenuate the glucose‐lowering effect of exogenous ketosis using a ketone ester (KE).

**Methods:**

This crossover study involved 10 healthy human volunteers who fasted for 24 h prior to the ingestion of 25 g of d‐β‐hydroxybutyrate (βHB) in the form of a KE drink (ΔG^®^) on two separate visits. During one of the visits, participants additionally ingested 2 g of L‐alanine to see whether L‐alanine supplementation would attenuate the glucose‐lowering effect of the KE drink. Blood L‐alanine, L‐glutamine, glucose, βHB, free fatty acids (FFA), lactate and C‐peptide were measured for 120 min after ingestion of the KE, with or without L‐alanine.

**Findings:**

The KE drinks elevated blood βHB concentrations from negligible levels to 4.52 ± 1.23 mmol/L, lowered glucose from 4.97 ± SD 0.39 to 3.77 ± SD 0.40 mmol/L, and lowered and L‐alanine from 0.56 ± SD 0.88 to 0.41 ± SD 0.91 mmol/L. L‐alanine in the KE drink elevated blood L‐Alanine by 0.68 ± SD 0.15 mmol/L, but had no significant effect on blood βHB, L‐glutamine, FFA, lactate, nor C‐peptide concentrations. By contrast, L‐alanine supplementation significantly attenuated the ketosis‐induced drop in glucose from 28% ± SD 8% to 16% ± SD 7% (*p* < .01).

**Conclusions:**

The glucose‐lowering effect of acutely elevated βHB is partially due to βHB decreasing L‐alanine availability as a substrate for gluconeogenesis.

## Graphical abstract

This was crossover study involved two separate visits for 10 healthy human volunteers who fasted for 24 h prior to the ingestion of 25 g of d‐β‐hydroxybutyrate (βHB) monoester (ΔG®). During one of the visits, participants ingested 2 g of L‐alanine. Blood L‐alanine, L‐glutamine, glucose, βHB, free fatty acids (FFA), lactate and C‐peptide were measured. Demonstrating that ketosis induces hypoglycemia, in part, by limiting L‐alanine availability for gluconeogenesis.

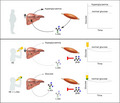

## 1 INTRODUCTION

In the fed state, the human brain relies almost exclusively on glucose as an energy source. However, in the fasted state or during carbohydrate scarcity, the human body has two adaptive mechanisms: (i) Gluconeogenesis, which promotes the catabolism of muscle tissue and (ii) Ketogenesis, which produces primarily d‐β‐hydroxybutyrate (βHB) from fat stores and provides an alternative fuel substrate for the brain. This latter option fuels the energy expensive brain, while at the same time preserving valuable lean tissue.^1^

Interestingly, L‐alanine is the amino acid with the largest concentration changes during prolonged starvation^2^ and the infusion of ketone bodies lowers L‐alanine blood levels more than any other amino acid.^3^ In fact, L‐alanine acts as an intermediary metabolite in the transfer of pyruvate from the skeletal muscle to the liver^4^ and, while it is far from being the most prominent gluconeogenic substrate, is particularly relevant for hepatic gluconeogenesis.^5^

On the contrary, in type 2 diabetes (T2D), excessive gluconeogenesis underlies hyperglycemia.^6^, ^7^, ^8^ Thus, one could predict that βHB, by inhibiting gluconeogenesis,^9^ could help manage hyperglycemia in T2D. Consistent with this hypothesis, ketogenic diets have been shown to effectively reverse T2D^10^, ^11^ and, while most of the benefit of ketogenic diets in T2D likely comes from carbohydrate reduction, it is not yet clear to what degree βHB itself might play a supporting therapeutic role. In fact, in both animals and humans, exogenous βHB are alone sufficient to decrease blood glucose.^12^, ^13^, ^14^, ^15^

Even further, it has been known for decades that ketone bodies reduce blood glucose even in people uncapable of producing insulin and that L‐alanine is likely involved in this mechanism.^3^

On this regard, indirect evidence suggests that ketone bodies limit the secretion of L‐alanine by reducing skeletal muscle glycolysis and, consequently, by limiting the availability of intramuscular pyruvate.^3^, ^16^

The development of an enantiomerically pure medical‐grade exogenous ketone ester (KE) for human consumption, following on the possibility that βHB could itself benefit persons with T2D, has inspired interest in the exploration of exogenous ketosis for T2D.^17^ Thus, it is plausible that exogenous ketones decrease blood glucose by reducing L‐alanine availability for gluconeogenesis and that this effect could help improve glucose control.^18^ This study sought to test this hypothesis by investigating whether L‐alanine supplementation attenuates the glucose‐lowering effect of exogenously induced ketosis.

## 2 METHODS

### 2.1 Ethics

This study was pre‐registered at ISRCTN16169021 and received Ethics approval by the East of England—Cambridge South Research Ethics Committee on 18 February 2019 (Reference 18/EE/0115). All participants gave their written informed consent.

This study was conducted in accordance with the guidelines set forth by the International Conference on Harmonisation Guidelines for Good Clinical Practice and the Declaration of Helsinki.^19^ Potentially pregnant participants were excluded.

### 2.2 Statistical analysis

We based our expected glucose difference (0.9 mmol/L) in what was reported as the maximal glucose difference between low and high doses of exogenous ketones in a similar setting.^15^ Sample size was calculated to detect a blood glucose difference of at least 0.9 mmol/L between two groups with eight measurements, and f effect size of 0.5 from a standard deviation of 0.9 within each group (measured but not reported in the final publication^15^), a statistical power of 80% and an alpha probability of 0.05. As a result, 10 participants per group were needed. To achieve a sex balanced cohort, five men and 5 women were recruited, and all completed the study. Sample size calculations were performed using G*Power (*F* tests – ANOVA: Repeated measures, between factors) version 3.1.^20^

All statistical tests and analysis were performed using Excel^®^ (Microsoft). Data, presented as means ± standard deviations (SD), were analysed using a trial × time, repeated measures ANOVA with replication and was adjusted with Bonferroni's correction for multiple comparisons. Differences were considered significant at *p* < .006 (equivalent to 0.05/8 after a Bonferroni correction for keeping a 0.05 significance threshold). Since baseline blood concentrations of L‐alanine and L‐glutamine have large interpersonal variations due to body composition, comparisons were made using the percentual change from baseline.

### 2.3 Materials and procedures

10 healthy participants engaged in a randomized crossover experiment in which they came to the laboratory on two occasions after a 24 h fast. On one visit, participants drank a mix 25 g of the ΔG^®^ KE ((R)‐3‐ hydroxy butyl (R)‐3‐hydroxybutyrate, provided by TdeltaS Ltd.) diluted in 25 ml water. On the other visit, participants consumed the same drink with an additional 2g of L‐alanine (provided by Hard Eight Nutrition LLC) to match skeletal muscle release of L‐alanine per 2 h.^6^ After the ingestion of the drinks, all subjects were asked to remain seated or laying in a semi‐Fowler position. They were allowed to perform quiet activities such as reading or watching movies.

After ingestion, the KE monoester bond is cleaved by esterases in the gut wall, yielding βHB and butanediol in equal amounts. Both are absorbed into the portal circulation and the latter is taken up by the liver, where it is converted to βHB by alcohol dehydrogenase. βHB leaves hepatocytes via monocarboxylate transporters. Pharmacokinetic studies have shown that, in the fasted and resting states, the βHB monoester can induce ketosis for 3–4 h, with a peak at ~1 h.^21^

Blood samples were collected through a venous cannula at baseline and for 120 min. After collection, samples kept on ice were centrifuged for 10 min at 2,897.86 *g*. The plasma component was aliquoted into Eppendorf tubes, and these were stored at −80°C until later analysis. Frozen samples were thawed at room temperature for ≈30 min before biochemical analyses were undertaken.

Glucose, βHB, lactate and free fatty acids were assayed using a commercial semi‐automated bench‐top analyser (ABX Pentra). L‐alanine, L‐glutamine and human C‐peptide were measured using kits (by Abcam: ab83394, ab145659, and ab178641, respectively).

## 3 RESULTS

### 3.1 Participant anthropometric characteristics

Ten healthy participants, five males and five females, were enrolled in this crossover trial. All participants were of normal weight (mean BMI 23.2 kg/m^2^ ± 2.4), and mean age was 40 years ± 16 years. Two of our five female participants were postmenopausal. One of the remaining three had been using a hormonal intrauterine device for the last 18 months at the time of enrolment, one had been using a subdermal contraceptive implant for the last 6 months at the time of enrolment and both visits of the other female participant happened during her early follicular phase.

Table 1 summarizes anthropometric characteristics of our participants. Ingestion of the KE drink was well‐tolerated by all participants and none presented symptomatic hypoglycaemia.

**TABLE 1:** Participant characteristics

Sex	5 (F), 5 (M)
Age (years)	40 (16) range: 19–70
Height (cm)	176 (15) range: 154–209
Weight (kg)	73 (18) range: 49–105
BMI (kg/m^2^)	23.2 (2.4) range: 19–27

Note

Data are expressed as mean (Standard deviation).

### 3.2 Blood L‐alanine, L‐glutamine and glucose concentration changes after ketone ester ingestion, with and without L‐alanine supplementation

In the absence of L‐alanine supplementation, inducing acute ketosis with the KE raised blood βHB concentration to 4.52 ± 1.24mmol/L and L‐glutamine by 9% ± 4% (absolute change from 1.09 to 1.22 ± 0.04 mmol/L).

By contrast, exogenous ketosis lowered blood L‐alanine by 26% ± 9.3% (absolute change from 0.56 to 0.44 ± 0.05 mmol/L) and blood glucose by 28% ± 8% (absolute change from 4.97 to 3.77 ± 0.41 mmol/L). Compared to baseline, changes in βHB, glucose and alanine, but not glutamine, were significantly different (*p* < .01).

In the presence of L‐alanine supplementation, inducing ketosis raised blood βHB concentration to 4.64 ± 1.35 mmol/L and L‐glutamine by 10% ± 6% (absolute change from 1.12 to 1.26 ± 0.05 mmol/L). The intra‐assay coefficient of all samples was <10%.

**FIGURE 1:**
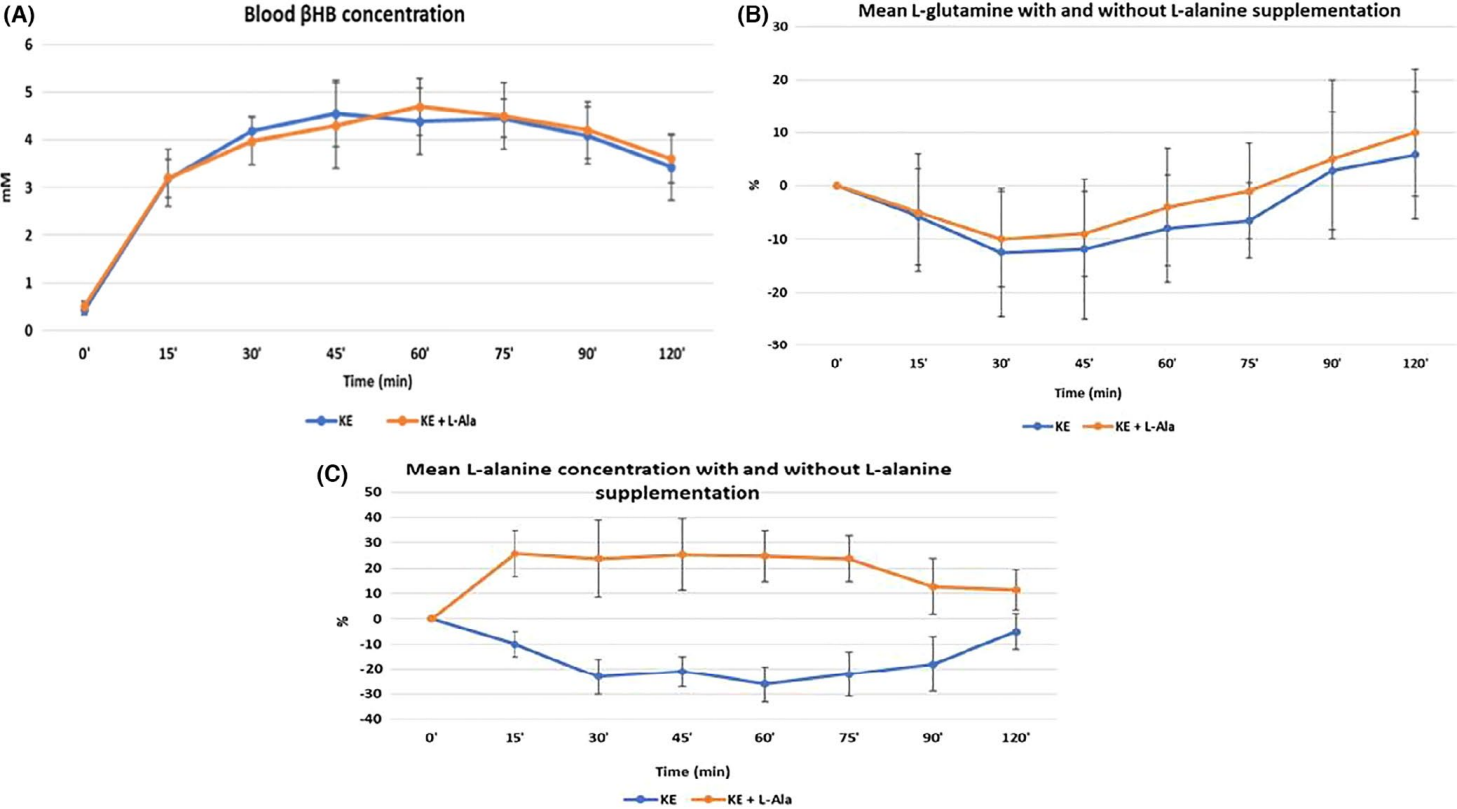
Blood L‐alanine, L‐glutamine and βHB concentration changes after inducing acute ketosis with and without L‐alanine supplementation. Data are presented as the mean ± SD (*n* = 10 participants, measurements by triplicate). As expected, Alanine supplementation raised blood Alanine throughout the trial

As expected, L‐alanine supplementation led to a net increase in L‐alanine by 16% (absolute change from 0.54 to 0.68 ± 0.05 mmol/L), thus maintaining L‐alanine availability for gluconeogenesis (Figure 1). Correspondingly, blood glucose decreased by a lesser amount, only by 17% ± 5% (absolute change from 5.01 to 4.03 ± 0.32 mmol/L). Again, changes in βHB, glucose and L‐alanine, but not L‐glutamine, were significant (*p* < .01).

### 3.3 L‐alanine supplementation attenuated the glucose‐lowering effect of acute ketosis

**FIGURE 2:**
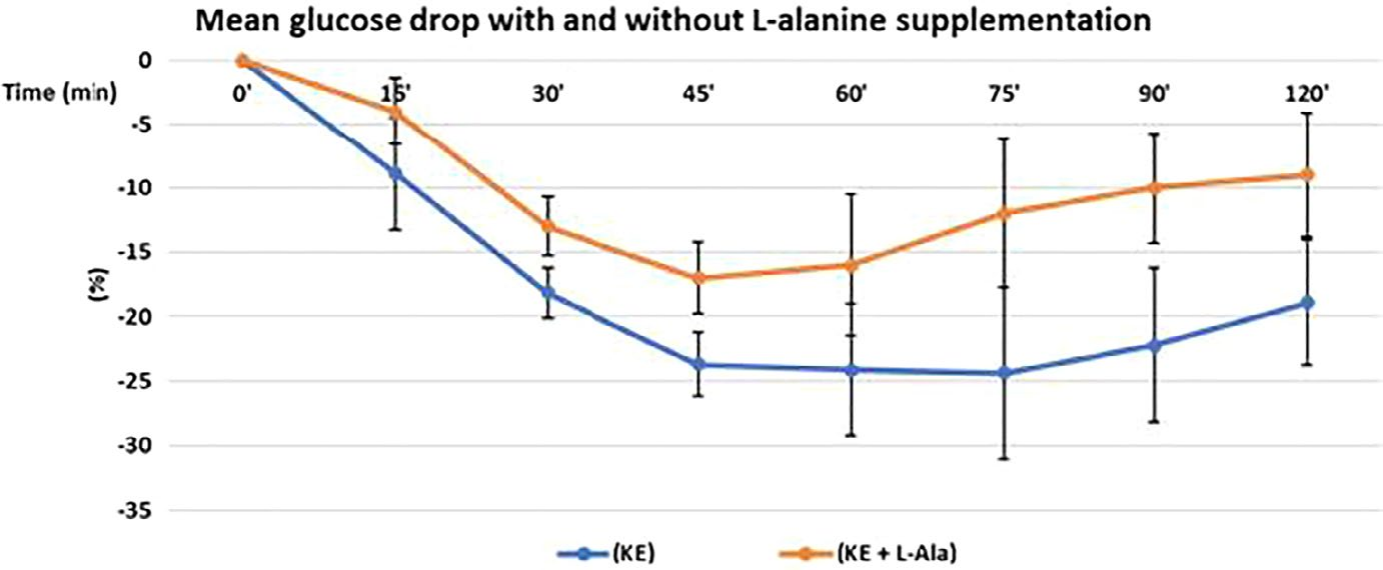
L‐alanine supplementation attenuated the glucose‐lowering effect of ketosis. Data are presented as mean concentration changes (*n* = 10 participants, measurements by triplicate). *Y*‐axis = per cent change from baseline of blood glucose concentration. *X*‐axis = Time (minutes) after drinking 25 of βHB ketone monoester. Mean change after baseline was analysed with a two‐way ANOVA (trial × time) and found to be statistically significant between treatments (KE vs. KE + L‐alanine) statistically significant differences (*p* < .006). Glucose data were tested for normality using a Shapiro‐Wilk test

2 g L‐alanine significantl reduced the magnitude of the glucose‐lowering effect of exogenous ketosis and glucose levels were consistently lower at all time points when L‐alanine was supplemented (Figure 2).

### 3.4 L‐alanine supplementation did not impact changes in blood lactate, free fatty acids (FFA) and C‐peptide during acute ketosis

**FIGURE 3:**
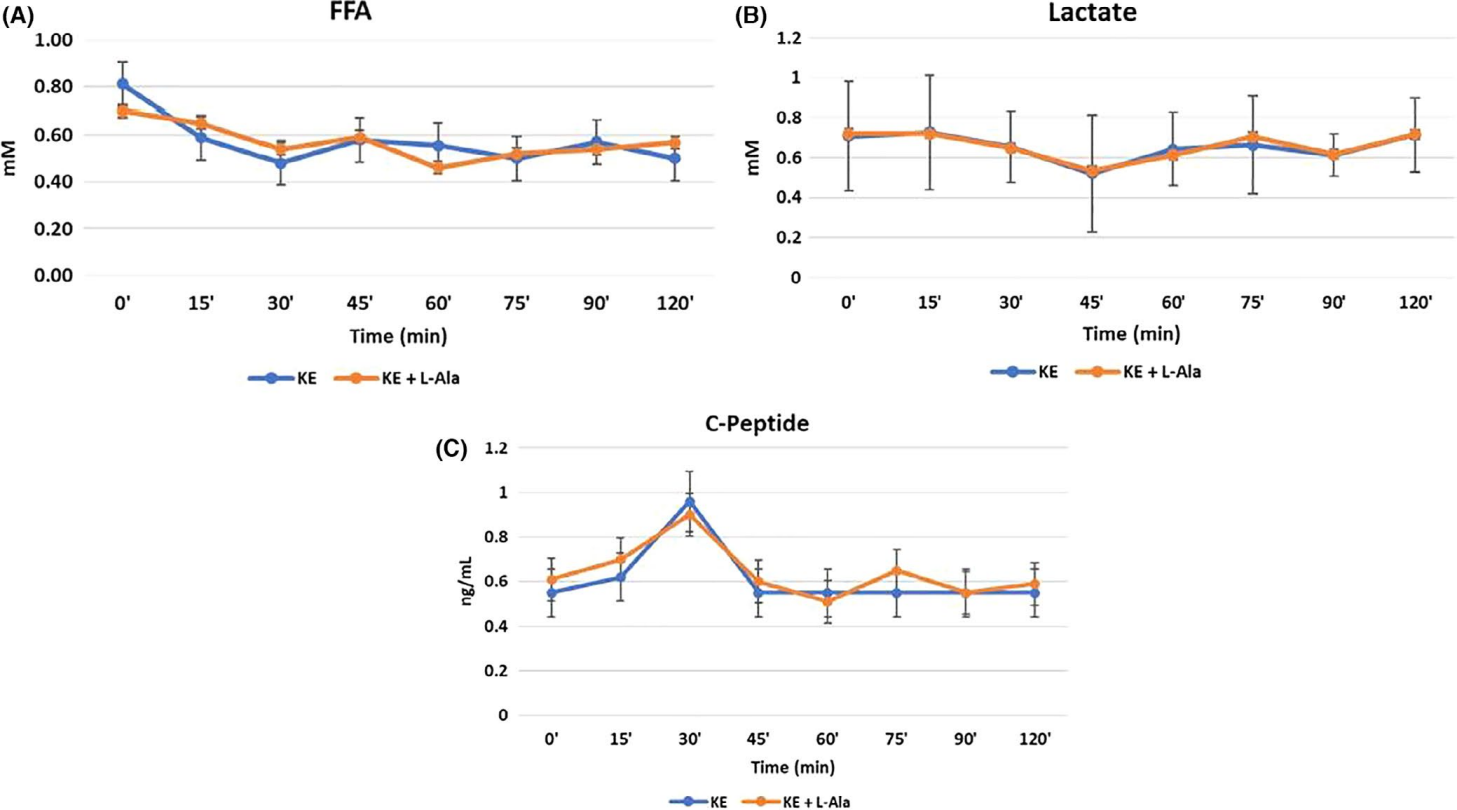
Blood lactate, free fatty acids (FFA) and C‐peptide during acute ketosis with and without L‐alanine. Mean change after baseline was analysed with a two‐way (trial × time) ANOVA. Statistical significance after a Bonferroni correction was defined as *p* < .006

Compared to baseline, FFA concentrations were significantly lower at most time points 30 min after KE ingestion. C‐peptide was moderately elevated in both groups at the 30 min time point. Blood lactate, FFA and C‐peptide concentrations were similar at all time points after ingestion of KE regardless of L‐alanine supplementation, as in Figure 3.

### 3.5 Correlation between the dose of ketone monoester with maximal βHB concentration and glucose change in the blood

Since all participants consumed 25 ml of the ketone monoester, they differed in the effective dose of ketone monoester they received, ranging between 242 and 491 mg/kg. As expected, βHB maximal blood concentration correlated with the dose of ketone monoester (*R* ^2^ = .61 *p* < .01). Interestingly, the correlation between the magnitude of the glucose change with the dose of ketone monoester was very comparable (*R* ^2^ = .59 vs *R* ^2^ = .61, respectively) suggesting changes in blood glucose were proportional to and mostly driven by the consumption of the ketone monoester. Figure [Link], [Link] illustrates these results.

## 4 DISCUSSION

These data suggest that a decrease in L‐alanine availability contributes to the glucose‐lowering effect of acute ketosis. However, it is also clear that the glucose‐lowering effect of acute ketosis is a result of multiple mechanisms.

### 4.1 The glucose‐alanine cycle

During prolonged fasting, L‐alanine blood levels decrease more than those of any other amino acid, largely because of hepatic uptake of L‐alanine to fuel gluconeogenesis.^2^ This phenomenon prompted the discovery of the glucose‐alanine cycle, in which pyruvate is transformed into L‐alanine (pyruvate in disguise) via transamination in skeletal muscle. L‐alanine is released to the bloodstream, taken up by the liver, and transformed back into pyruvate to fuel gluconeogenesis.^4^ Thus, any reduction in intramuscular pyruvate would result in lower L‐alanine levels in the blood and less gluconeogenesis.

Ketone oxidation decreases intramuscular pyruvate via multiple mechanisms: (i) First, ketone oxidation reduces glycolysis and pyruvate in skeletal muscle^16^ and (ii) second, ketone body oxidation increases acetyl CoA levels.^22^ Acetyl CoA is an allosteric activator of pyruvate carboxylase, thus, driving the conversion of pyruvate to oxaloacetate.^23^ (iii) In addition to decreasing pyruvate for transamination into L‐alanine, ketosis also directly decreases L‐alanine production by reducing skeletal muscle protein breakdown.^24^

Furthermore, in the liver, L‐alanine supports gluconeogenesis. L‐alanine allosterically inhibits the liver isozyme of pyruvate kinase, the enzyme responsible for the last step of glycolysis.^25^ Thus, L‐alanine restriction would favour glycolysis over gluconeogenesis. L‐alanine is also a potent glucagon secretion agonist,^26^ and glucagon stimulates gluconeogenesis. Even further, the blood concentration of glucagon also drops after the ingestion of βHB salts after an overnight fast;^27^ however, it does not in the postabsorptive state.^28^

### 4.2 The glucose‐lowering effect of ketosis is context dependent and pleiotropic

As observed in this study, the ability of exogenously induced ketosis to reduce circulating L‐alanine as fuel for gluconeogenesis cannot account for the entire glucose‐lowering effect of KE ingestion. Other mechanisms are at play.

Infusion and islet studies have shown that exogenous ketosis can stimulate insulin secretion.^29^, ^30^ However, even while we observed a transient C‐peptide spike at 30 min after KE ingestion, it should be noted that this does not necessarily translate into an equal magnitude, one‐to‐one, spike in systemic circulating insulin due to insulin liver extraction. What is more, even if we assume this C‐peptide change correlates with an equal magnitude insulin change, the latter would not be large or long enough to account for the entire glucose‐lowering effect observed.

There is also evidence that a decrease in gluconeogenesis and hepatic glucose output, rather than an increase in insulin‐mediated glucose clearance from the blood, accounts for most of the glucose‐lowering effect of ketosis.^9^ For example, exogenous ketone infusions in patients with type 1 diabetes demonstrate that acute ketosis lowers blood glucose^3^ even in the absence of insulin. Thus, it is unlikely that the minor insulinogenic effect of exogenous ketosis accounts for most of the glucose‐lowering effect KE ingestion.

The glucose‐lowering effect of acute ketosis is also not restricted to the fasting state. Inducing acute ketosis lowers the postprandial glycaemic curve after a dextrose challenge without inducing significant differences in insulin secretion.^31^ One of the proposed mechanisms is that βHB‐mediated inhibition of lipolysis^31^ depletes the blood supply of fatty acids, driving an increase in glucose uptake. However, this has not been observed after the ingestion of niacin, which also blocks lipolysis by acting on the same receptor as βHB and has no effect on glycaemia.^32^

Additionally, as mentioned before, it is also worth highlighting that acute ketosis lowered glucose in postabsorptive humans despite not lowering glucagon.^28^ As gluconeogenesis contributes to elevations in blood glucose in the postabsorptive state, βHB‐induced attenuation of gluconeogenesis could also explain (at least partially) the postprandial attenuation of the glycaemic curve that has been previously observed.

It is also worth noting that glycerol is a gluconeogenic substrate. However, evidence suggests that, in the fasting humans, L‐alanine, and lactate, not glycerol, are the major gluconeogenic substrates. Additionally, limiting FFA availability could also contribute to the observed glucose‐lowering effect of KEs by reducing β‐oxidation and, therefore, the availability of reducing equivalents, high energy phosphates and acetyl‐CoA (which, as mentioned before, allosterically regulates pyruvate carboxylation^23^). Nonetheless, since ketosis also reduces the availability of glycerol and FFA by inhibiting lipolysis,^33^ ketosis could be potentially beneficial for individuals in whom gluconeogenesis is pathologically elevated^27^ regardless of which substrate is the most important contributor to gluconeogenesis.

Importantly, it is unlikely that this is a KE‐specific effect. While the hepatic conversion of butanediol derived from ketone ester into βHB would alter hepatocyte NAD+/NADH balance (therefore altering endogenous glucose production), studies comparing the ingestion of equimolar quantities of ketone salts and ketone esters (that lack a butanediol component) demonstrate glucose‐lowering effects of similar magnitudes.^15^

In contrast, a study in people with type 2 diabetes and using sodium acetoacetate infusions with and without somatostatin concluded that ketone bodies can suppress elevated hepatic glucose output independently of changes in insulin secretion, only when the concomitant stimulation of glucagon secretion is prevented.^34^

Even when the physiological effects of exogenous acetoacetate are not comparable to those of exogenous βHB (because they have opposite effects on the NAD/NADH couple), since alanine is a potent glucagon secretion agonist, βHB‐induced reduction would also yield less glucagon secretion.

**FIGURE 4:**
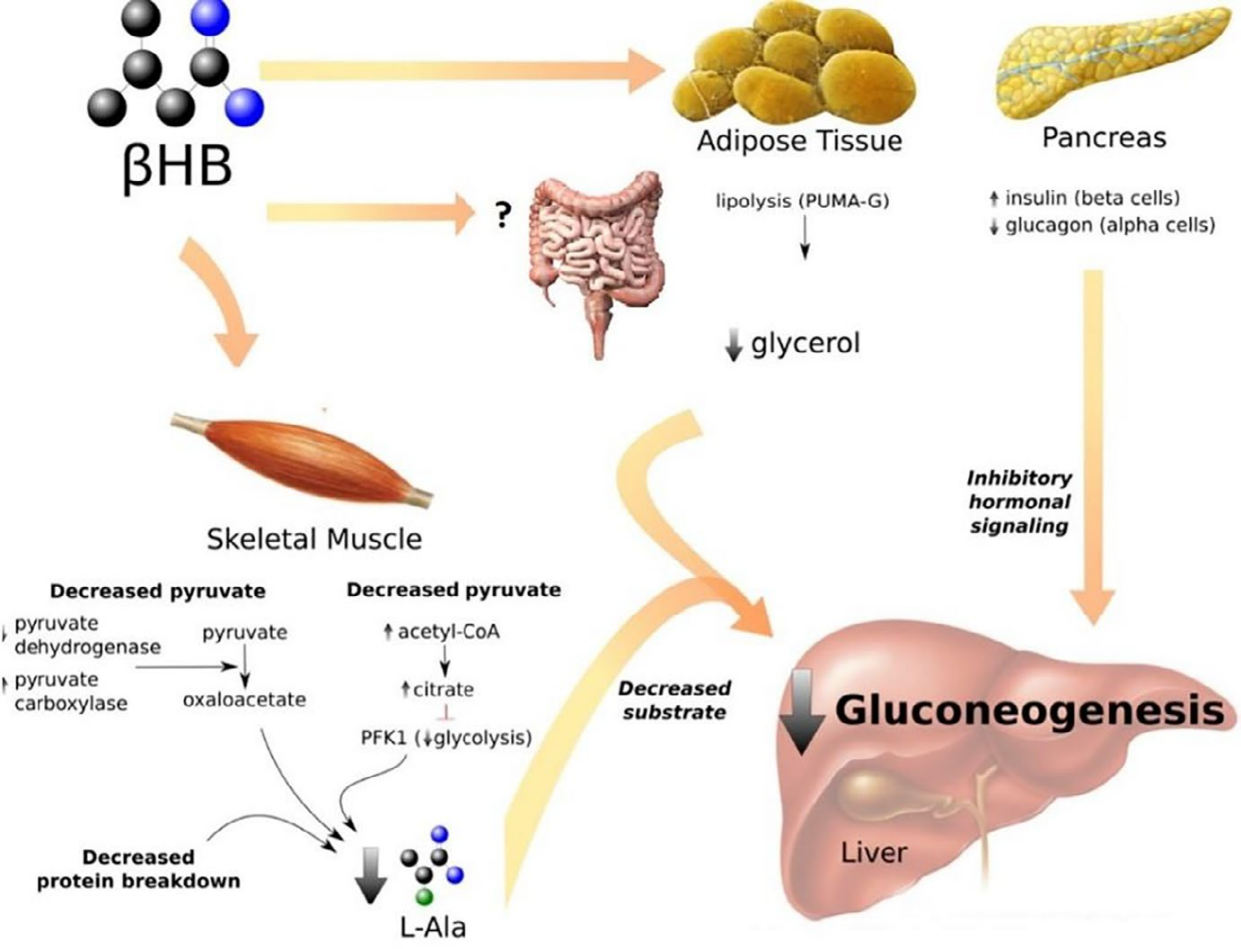
Mechanisms whereby ketosis lowers blood glucose. In skeletal muscle: βHB increases the concentration of acetyl CoA,^22^ which inhibits pyruvate dehydrogenase and activates pyruvate carboxylase.^23^ As a result, more pyruvate is transformed into oxaloacetate. Additionally, the rise in acetyl CoA inhibits phosphofructokinase‐1 (PFK1), downregulating glycolysis and, therefore, decreasing pyruvate production.^45^ Through these two mechanisms, pyruvate levels are reduced and there is less pyruvate available for transamination into L‐alanine. βHB also decreases protein breakdown, further reducing L‐alanine release.^16^ In adipose tissue: βHB inhibits lipolysis via de PUMA‐G receptor,^33^ reducing the release of glycerol, a minor gluconeogenic substrate. In the pancreas: βHB promotes insulin release by the beta cells.^30^, ^46^ Furthermore, there is less L‐alanine to stimulate glucagon release by the alpha cells.^26^ The decrease in gluconeogenic substrates (L‐alanine and glycerol) and inhibitory hormonal signalling (increase in insulin/glucagon ratio; and L‐alanine allosterically inhibits pyruvate kinase^25^) cause a decrease in gluconeogenesis by the liver (and also possibly other peripheral tissues, like the intestines, that can perform gluconeogenesis). The net result of acute ketosis is a small increase in peripheral glucose uptake, due to a small increase in insulin, and a larger decrease in glucose release by gluconeogenic tissues

Figure 4 summarizes some of the mechanisms whereby exogenously induced acute ketosis could lower blood glucose concentration.

### 4.3 Limitations of this study

First, the relative contribution of the different gluconeogenic organs (liver, kidneys and intestine) and of the different gluconeogenic substrates (lactate, glycerol, alanine and glutamine) varies depending on whether a person is in the fed or fasted state.^35^, ^36^ Since we hypothesized Alanine's role was due to its potential influence on gluconeogenesis, we chose to evaluate the effects of acute ketosis after fasting for 24 h because the relative contribution of gluconeogenesis to endogenous glucose production is 47 ± 49% after 14 h and 67 ± 41% after 22 h.^37^, ^38^ However, there is evidence suggesting that, in the context of short‐term fasting, alanine is the most important contributor via liver gluconeogenesis.^5^, ^39^, ^40^, ^41^, ^42^

Additionally, our sample size is likely underpowered to detect specific time point differences between both interventions. However, finding these difference falls beyond the scope of our work since we are interested in the overall effect of Alanine on the glucose reduction curve and not so much in its effect on a particular time point along that curve.

Since all participants in this study were fasted for control purposes, we cannot extrapolate these insights to other metabolic states where there are different relative contributions from the kidneys and the intestines. Future studies may choose to investigate the same question in postprandial participants or using labelled substrates.

Additionally, while unlikely, an unexpected glucose‐lowering interaction effect between L‐alanine supplementation and exogenously induced ketosis cannot be entirely ruled out. However, other studies have observed the opposite (an increment in hepatic glucose production and ketosis downregulation after Alanine supplementation).^43^ Nonetheless, a future study should include an independent L‐alanine arm to compare the impact of L‐alanine on glycaemia in the absence of ketosis. Additional limitations (since we did not measure them) and informative measurements for future studies are nitrogen balance and glucagon.

Finally, it has been proposed that glycerol is the most relevant gluconeogenic substrate.^44^ Even if this is true, since acute ketosis also reduces the availability of glycerol, the overall conclusion of ketosis being potentially therapeutic for individuals in whom gluconeogenesis is pathologically elevated holds true.

## 5 CONCLUSION

In fasting healthy humans, exogenous ketosis results in a reduction in L‐alanine availability to fuel gluconeogenesis and that this contributes, at least partially, to the glucose‐lowering effects of acute ketosis. These data provide insight about the potential mechanisms behind the elsewhere reported benefits of ketogenic interventions for patients living with type 2 diabetes. More research is needed to confirm these findings.

## CONFLICT OF INTERESTS

The intellectual property covering the uses of ketone bodies and ketone esters are owned by BTG Plc, Oxford University Innovation Ltd and the US National Institutes of Health. Professor Kieran Clarke, as an inventor, will receive a share of the royalties under the terms prescribed by each institution. Professor Clarke is a director of TdeltaS Ltd, a company spun out of the University of Oxford to develop products based on the science of ketone bodies in human nutrition. The other authors declare that they have no competing financial interests or personal relationships that could have influenced the work reported in this paper.

## AUTHOR CONTRIBUTION

**Adrian Soto‐Mota:** Conceptualization (lead); Data curation (lead); Formal analysis (lead); Investigation (lead); Methodology (lead); Software (lead); Visualization (lead); Writing‐original draft (lead). **Nicholas G. Norwitz:** Formal analysis (supporting); Visualization (supporting); Writing‐original draft (supporting). **Rhys D. Evans:** Conceptualization (supporting); Formal analysis (supporting); Investigation (supporting); Methodology (supporting); Supervision (equal); Writing‐original draft (equal). **Kieran Clarke:** Conceptualization (equal); Formal analysis (equal); Investigation (equal); Methodology (equal); Resources (equal); Supervision (equal); Writing‐original draft (equal).

## Supporting information

Fig S1A

Fig S1B

## Data Availability

Data described in the manuscript, code book and analytic code will be made available upon request pending approval by TdeltaS Ltd.
