# Supplementary material for: Exogenous d‐β‐hydroxybutyrate lowers blood glucose in part by decreasing the availability of L‐alanine for gluconeogenesis
Source: Endocrinol Diabetes Metab. 2021 Nov 16;5(1):e00300. doi: 10.1002/edm2.300 (PMC8754249; doi:10.1002/edm2.300)
Supplement: Supplementary file 2 [file EDM2-5-e00300-s002.rtf]

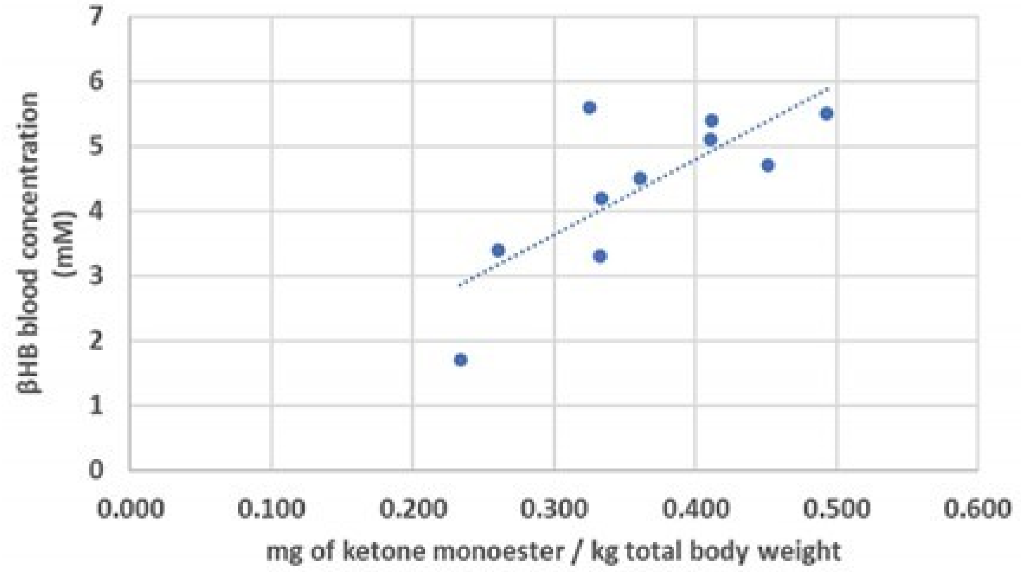

βHB blood concentration (mM)
7
6
5
4
3
2
1
0
0.000
0.100
0.200
0.300
0.400
0.500
0.600
mg of ketone monoester / kg total body weight
